# Microbial Etiology of Postoperative Spinal Fusion Infections and Factors Associated with Gram-Negative Infections, United States, 2021–2022

**DOI:** 10.3201/eid3211.260662

**Published:** 2026-11

**Authors:** Molly Lindstrom, Benjamin W. Langworthy, Jonathan N. Sembrano, Don Bambino Geno Tai

**Affiliations:** University of Minnesota Medical School, Minneapolis, Minnesota, USA (M. Lindstrom, J.N. Sembrano, D.B.G. Tai); University of Minnesota School of Public Health, Minneapolis (B.W. Langworthy)

**Keywords:** bacteria, surgical site infections, spinal fusion, gram-negative infection, National Healthcare Safety Network, healthcare-associated infections, infection prevention, microbiology, cause of infection, spinal instrumentation infection, spinal infection, United States

## Abstract

Accurate knowledge of microbial etiology is essential for optimizing spinal fusion surgical site infection (SSI) management, yet contemporary national data in the United States, particularly prevalence and factors associated with gram-negative infections, remain poorly understood. We conducted a cross-sectional analysis of US data reported to the Centers for Disease Control and Prevention National Healthcare Safety Network (2021–2022) to characterize the microbiology of spinal fusion SSI and identify factors associated with gram-negative infection. Among 7,187 cases, gram-negative organisms caused 31.5% (n = 2,265) of SSIs. Older age, female sex, higher body mass index, longer operative times, higher American Society of Anesthesiologists class, lumbar involvement, inpatient surgery, and emergent and trauma surgeries were independently associated with gram-negative infections relative to monomicrobial *Staphylococcus aureus* infections. Our findings might inform future studies focusing on high-risk cohorts as targets for expanded gram-negative prophylactic regimens. Clinicians can use risk profiles to guide empiric gram-negative antibiotic coverage in treating SSIs.

Surgical site infection (SSI) after spinal surgery occurs in ≈2% of procedures and is associated with substantial illness and healthcare utilization (*1*,*2*). Evidence suggests that the percentage of gram-negative pathogens in spinal fusion SSIs is higher than for other musculoskeletal infections (*3*). However, whether that trend persists across a large-scale national cohort remains unverified. Furthermore, the specific independent factors for gram-negative involvement among patients with spinal fusion SSIs remain poorly understood. Although institution-specific studies offer valuable insights, their findings are inherently susceptible to local surgical practices, institutional protocols, and regional healthcare epidemiology, thereby limiting their generalizability.

Our objective in this study was to characterize the contemporary national distribution of microbial etiology in spinal fusion SSI. We also aimed to identify the patient characteristics or procedural characteristics that are associated with gram-negative infection in spinal fusion SSI.

## Methods

We performed a cross-sectional study of SSI episodes associated with spinal fusions in the United States reported to the Centers for Disease Control and Prevention National Healthcare Safety Network (NHSN) during January 2021–December 2022. We acquired deidentified data through a Freedom of Information Act request. Spinal fusion infection cases were reported to the NHSN voluntarily by participating hospitals and surgical centers using the criteria for surgical site infection (*4*). We selected the 2021–2022 cohort because it was the most recently completed dataset available at the time of our data request, enabling us to evaluate the most contemporary trends.

We abstracted available patient demographic and clinical characteristics from the database. Those data consisted of age, sex, body mass index (BMI), diabetes status, American Society of Anesthesiologists (ASA) physical status classification, presence of trauma before surgery, emergency status, and timing of infection relative to the index spinal fusion. Extracted surgical and procedural characteristics consisted of operative approach (anterior, posterior, or combined), wound class at the time of the index procedure, spinal level, method of wound closure (primary vs. other), procedural setting (inpatient vs. outpatient), and use of a scope or percutaneous endoscopic approach. We excluded cases in which an infection was explicitly documented as present at the time of surgery, because those cases represent a distinct clinical entity from postoperative spinal fusion SSIs.

We defined the unit of analysis (denominator) at the case level rather than at the isolate level. For patients with multiple positive cultures during a single SSI episode, we compressed duplicate bacterial species isolated from the same case and counted them only once. To analyze the independent predictors of different microbiological profiles, we categorized cases into 3 mutually exclusive, hierarchically defined analytic groups on the basis of the identified organisms. We categorized any case in which *S. aureus* was isolated as the sole microbial etiology as monomicrobial *S. aureus* infection (the reference group). The category of gram-negative infection consisted of all cases in which >1 gram-negative bacterial organism was isolated, regardless of whether it occurred as a monomicrobial infection or as part of a mixed/polymicrobial infection (i.e., coisolated with gram-positive organisms). Last, we categorized any case in which >1 gram-positive bacterial species were isolated without the presence of any gram-negative bacteria, *S. aureus*, or *Enterococcus* species as other gram-positive infection. The third cohort served as an exploratory group to approximate the profile of normal cutaneous flora. We excluded cases that did not meet the criteria for those 3 analytic categories or culture-negative cases from the multinomial regression model (Figure). 

**Figure:**
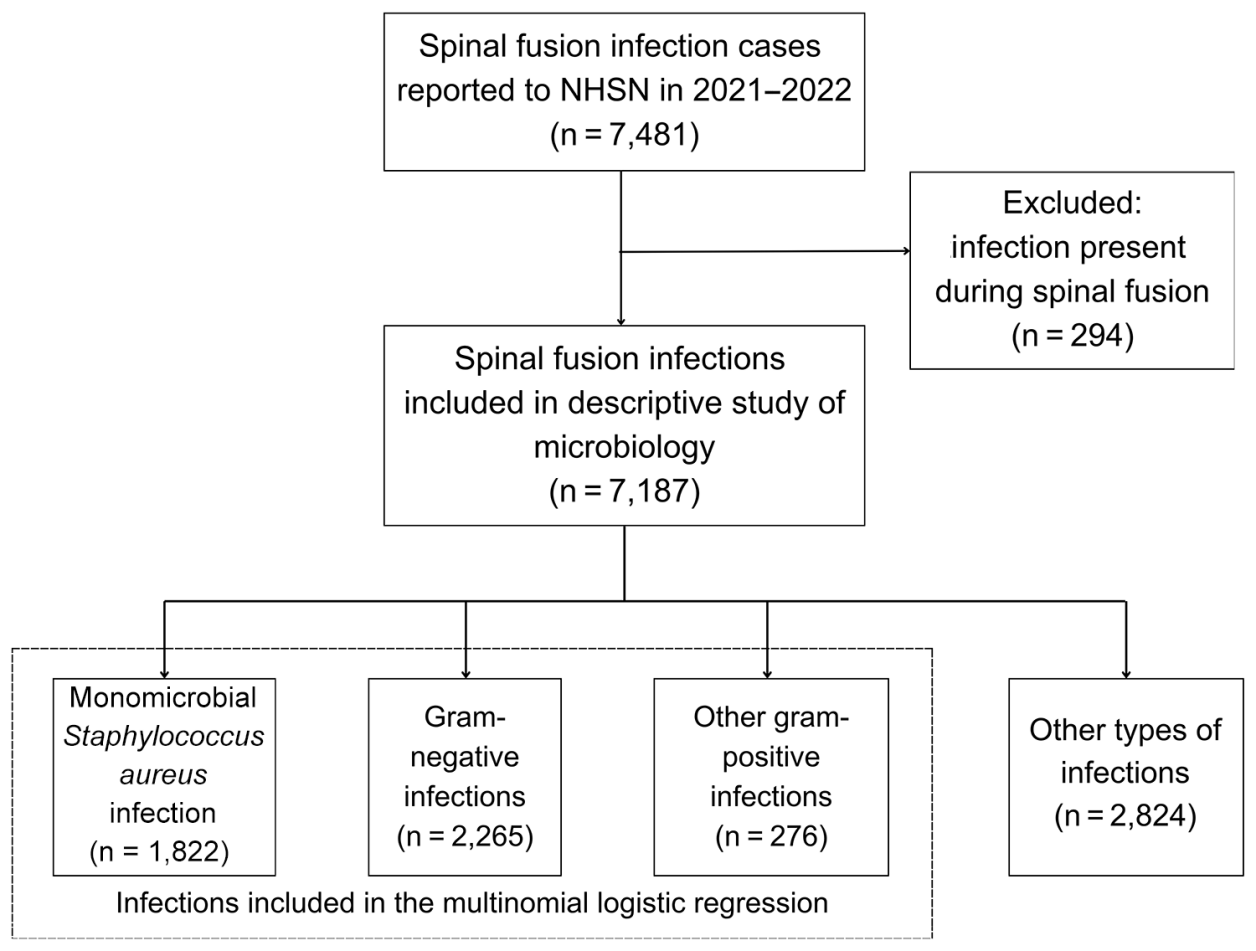
Flow diagram of postoperative surgical fusion infection cases in study of microbial etiology of postoperative spinal fusion infections and factors associated with gram-negative infections. NHSN, National Healthcare Safety Network.

### Statistical Analysis

We used frequency counts and percentages for categorical variables and used median (interquartile range [IQR]) or mean (SD) for continuous variables as appropriate. We performed a multinomial logistic regression analysis to determine the patient and procedural characteristics associated with the microbiological etiology of the infection. Our primary analysis evaluated the odds of a gram-negative infection compared with a reference group of monomicrobial *S. aureus* infection. To assess whether coinfection with other organisms influenced the observed effects, we performed a secondary analysis evaluating 2 additional cohorts within our model. The secondary analysis evaluated whether monomicrobial gram-negative and other gram-positive infections showed consistent results when compared with the same reference group. We followed that process to ensure the primary results were not driven by coinfections. The model simultaneously accounted for all patient and procedural covariates detailed above. We conducted analysis by using R version 4.3.2 (The R Project for Statistical Computing, httpw://www.r-project.org) and performed multinomial logistic regression using the multinom function in the nnet package. For multinomial logistic regression, the odds ratio (OR) can be interpreted as the multiplicative effect on the odds of infection relative to the reference group for a one-unit increase in continuous variable, or relative to the reference category for a categorical variable.

## Results

A total of 7,481 cases of spinal fusion SSI were reported to the NHSN during January 1, 2021–December 31, 2022. After excluding 294 (3.9%) cases because of the presence of infection during the initial fusion surgery, 7,187 cases remained for primary analysis. The mean patient age was 61.7 (SD 13.4) years; 54.1% were female (n = 3,889) and 45.9% (n = 3,297) were male (sex was unknown for 1 case). Most procedures were performed in an inpatient setting (n = 7,084 [98.6%]) and used a posterior surgical approach (n = 5,686 [83.7%]). The mean operative duration was 3.86 (SD 2.3) hours, and the median time from spinal fusion to infection diagnosis was 21 (IQR 14–29) days (Table 1).

**Table 1:** Demographics and clinical characteristics of patients with spinal fusion surgical site infections in study of microbial etiology of postoperative spinal fusion infections and factors associated with gram-negative infections, United States, 2021–2022*

Characteristic	All patients, n = 7,187
Mean age, y (SD)	61.67 (13.4)
Mean BMI (SD)	32.26 (7.8)
Sex	
M	3,297 (45.9)
F	3,889 (54.1)
Unknown	1
Outpatient	103 (1.4)
Mean duration of surgery, h (SD)	3.86 (2.3)
Approach	
Anterior	920 (13.5)
Posterior	5,686 (83.7)
Both	189 (2.8)
ASA class	
1	40 (0.6)
2	1,568 (23.1)
3	4,649 (68.4)
4	533 (7.9)
5	3 (0.04)
Diabetes	2,216 (32.6)
Wound class	
Clean	6,574 (96.8)
Clean-contaminated	135 (2.0)
Contaminated	23 (0.3)
Dirty	63 (0.9)
Spinal level	
Axis-atlas	4 (0.1)
Axis-atlas/cervical	60 (0.9)
Cervical	1,367 (20.1)
Cervical/dorsal/ dorsolumbar	300 (4.4)
Dorsal/dorsolumbar	1,299 (19.1)
Lumbar/lumbosacral	3,765 (55.4)
Trauma, yes	349 (5.1)
Closure	
Intentionally left open	65 (1.0)
Fully closed or partial	6,728 (99.0)
Timing of infection, days from procedure, median (IQR)	21 (14, 29)
Emergency	521 (7.7)
Use of scope	21 (0.3)

*Values are no. (%) except as indicated. ASA, American Society of Anesthesiologists; BMI, body mass index; IQR, interquartile range.

### Microbiology of Spinal Fusion SSI 

Most cases were monomicrobial infections (n = 4,407 [61.3%]), 1,263 (17.6%) cases were polymicrobial, and the rest were culture-negative (1,517 [21.1%]). A total of 7,551 unique microorganisms were isolated among all cases reported to NHSN during the study period. Gram-positive bacteria were implicated in 53% (n = 3,813) of all cases. *Staphylococcus* spp. was the most prevalent bacterial genus, infecting 42.4% (n = 3,044) of cases. *S. aureus* was identified as the single most common pathogen, isolated in 30.6% (n = 2,200) of all cases (Table 2; Appendix Table). Gram-negative bacteria were implicated in 31.5% (n = 2,265) of cases; the most common species were *Escherichia coli* (n = 565 [7.9%]) and *Pseudomonas aeruginosa* (n = 501 [7.0%]). Fungi and mycobacterial infections were very rare; <2% of cases involved fungal organisms, and 0.1% involved mycobacteria.

**Table 2:** Distribution of causative microorganisms in postoperative spinal infections after spinal fusion in study of microbial etiology of postoperative spinal fusion infections and factors associated with gram-negative infections, United States, 2021–2022*

Microorganism	No. (%) cases, n = 7,187
*Staphylococcus* species	3,044 (42.4)
* Staphylococcus aureus*	2,200 (30.6)
Coagulase-negative staphylococci (except *S. lugdunensis*)	824 (11.5)
* S. lugdunensis*	73 (1.0)
*Enterococcus* species	340 (4.7)
*Streptococcus* species, including nutritionally variant streptococci	191 (2.7)
Other commensal gram-positive bacteria (e.g., *Corynebacterium* species)	143 (2.0)
Enterobacterales	1,786 (24.9)
* Escherichia coli*	565 (7.9)
* Proteus mirabilis*	362 (5.0)
* Klebsiella pneumoniae*	297 (4.1)
* Enterobacter cloacae* complex	275 (3.8)
* Serratia marcescens*	255 (3.5)
Nonfermenting gram-negative bacilli	560 (7.8)
* Pseudomonas aeruginosa*	501 (7.0)
Other mucosal gram-negative bacilli (e.g., *Hemophilus* species)	16 (0.2)
*Cutibacterium* species	274 (3.8)
Other anaerobic bacteria (e.g., *Bacteroides*, *Finegoldia* species)	214 (3.0)
Fungi	139 (1.9)
* Candida* species	131 (1.8)
Mycobacteria	8 (0.1)
Organism not fully identified	144 (2.0)

*All organisms represent unique organisms identified in all cultures across cases. The total number of unique organisms (n = 7,512) is greater than the total number of reported cases (n = 7,187) due to the number of polymicrobial cases reported. Organism frequency is reported as the percent of organisms involved across cases. Enterobacterales category includes additional species not individually listed. Nonfermenting gram-negative bacilli include species other than *Pseudomonas aeruginosa*. Percentages do not sum to 100% because not all organisms are listed in this table.

### Variables for Types of Infections

In multivariable multinomial logistic regression comparing gram-negative infections to a reference group of monomicrobial *S. aureus* infections, several independent predictors demonstrated significantly increased odds of a gram-negative etiology. Female sex was associated with 39% (95% CI 1.19–1.59) higher odds of gram-negative infection (p<0.001). Increasing age was associated with higher odds of gram-negative infection (OR 1.03 [95% CI 1.02–1.03] per year; p<0.001), representing a 3% increase in the likelihood of gram-negative involvement per additional year of age. Higher BMI was similarly associated with an increased likelihood of gram-negative infection (OR 1.03 [95% CI 1.02–1.04]; p<0.001).

Each additional hour of operative duration increased the odds of a gram-negative infection by 20% (OR 1.20 [95% CI 1.16–1.24]; p<0.001). A greater comorbidity burden was also strongly and independently associated with higher odds of a gram-negative etiology in a stepwise manner; patients classified as ASA class 4 exhibited a nearly 16-fold increase in the odds of gram-negative infection compared with those in ASA class 1 (OR 15.7 [95% CI 3.35–73.5]; p<0.001). Furthermore, surgical procedures involving the lumbar spine (OR 1.51 [95% CI 1.23–1.86]; p<0.001) and cases associated with trauma (OR 1.91 [95% CI 1.38–2.64]; p<0.001) demonstrated significantly higher odds of manifesting a gram-negative infection rather than monomicrobial *S. aureus*.

A longer duration of time between the index procedure and diagnosis was associated with significantly lower odds of a gram-negative infection (OR 0.97 [95% CI 0.96–0.97] per day; p<0.001), corresponding to a 3% reduction in odds for each additional postoperative day. Patients who underwent outpatient procedures were also 59% less likely to develop gram-negative infections than were those in the reference group (OR 0.41 [95% CI 0.18–0.96]; p = 0.041) (Table 3).

**Table 3:** Multinomial logistic regression analysis of factors associated with gram-negative spinal infections in study of microbial etiology of postoperative spinal fusion infections and factors associated with gram-negative infections, United States, 2021–2022*

Characteristic	*Staphylococcus aureus* infection, N = 1,822	Gram-negative infection, n = 2,265		
		No. infections	OR (95% CI)	p value
Mean age, y (SD)	59.4 (13.6)	64.1 (13.5)	1.03 (1.02–1.03)	<0.001
Mean BMI (SD)	31.1 (7.6)	33.4 (8.1)	1.03 (1.02–1.04)	<0.001
Unknown	123 (6.8)	112 (4.9)	–	–
Male sex	862 (47.3)	884 (39.1)	0.72 (0.63–0.84)	<0.001
Unknown	–	1	–	–
Outpatient surgery	28 (1.5)	10 (0.4)	0.41 (0.18–0.96)	0.041
Duration of surgery, h	3.59 (2.1)	4.4 (2.5)	1.20 (1.16–1.24)	<0.001
Approach				
Anterior	208 (12.2)	204 (9.5)	Referent	
Posterior	1,447 (85.2)	59 (2.7)	1.01 (0.80–1.28)	>0.9
Both	44 (2.6)	1,890 (87.8)	0.87 (0.54–1.40)	0.6
Unknown	123 (6.8)	112 (4.9)	–	–
ASA class				
1	16 (0.9)	2 (0.1)	Referent	
2	465 (27.4)	369 (17.2)	4.90 (1.06–22.6)	0.042
3	1,131 (66.6)	1,556 (72.3)	7.15 (1.56–32.8)	0.011
4	86 (5.1)	224 (10.4)	15.7 (3.35–73.5)	<0.001
Unknown	123 (6.8)	113 (5.0)	–	–
Diabetes	492 (29.0)	757 (35.2)	1.07 (0.92–1.24)	0.4
Wound class				
Clean	1,656 (97.5)	2,064 (95.9)	Referent	
Clean-contaminated	28 (1.7)	54 (2.5)	1.74 (1.06–2.86)	0.030
Contaminated	4 (0.2)	12 (0.6)	2.14 (0.65–7.07)	0.2
Dirty	11 (0.7)	23 (1.1)	1.75 (0.79–3.85)	0.2
Unknown	123 (6.8)	112 (4.9)	–	–
Spinal level				
Axis-atlas/cervical	12 (0.7)	21 (0.7)	Referent	
Cervical/dorsal/dorsolumbar	332 (19.5)	330 (14.5)	1.11 (0.77–1.62)	0.6
Dorsal/dorsolumbar	350 (20.6)	469 (21.8)	1.27 (0.99–1.63)	0.056
Lumbar/lumbosacral	916 (53.9)	1,333 (61.9)	1.51 (1.23–1.86)	<0.001
Unknown	123 (6.8)	112 (4.9)		
Trauma	87 (5.1)	139 (6.5)	1.91 (1.38–2.64)	<0.001
Closure				
Intentionally left open	14 (0.8)	21 (0.98)	Referent	
Fully closed or partial	1,684 (99.2)	2,131 (99.0)	1.08 (0.50–2.33)	0.9
Unknown	123 (6.8)	112 (4.9)		
Timing of infection, mean days from procedure (IQR)	24 (17–33)	19 (14–27)	0.97 (0.96–0.97)	<0.001
Depth of infection				
Superficial	424 (23.3)	657 (29.0)	Referent	
Deep	868 (47.6)	1,025 (45.3)	0.88 (0.74–1.05)	0.15
Organ space	530 (29.1)	583 (25.7)	0.85 (0.70–1.03)	0.1
Emergency	120 (7.1)	167 (7.8)	0.99 (0.75–1.32)	>0.9
Unknown	123 (6.8)	112 (4.9)	–	–
Use of scope	2 (0.1)	7 (0.3)	2.60 (0.51–13.4)	0.3
Unknown	123 (6.8)	112 (4.9)	–	–

*Values are no. (%) except as indicated. ORs represent the relative odds of gram-negative infection compared with the reference group of monomicrobial *Staphylococcus aureus* spinal fusion surgical site infection. ASA level 5 was removed because of insufficient numbers. Dashes indicate value was not calculated. ASA, American Society of Anesthesiologists; IQR, interquartile range; OR, odds ratio.

For monomicrobial gram-negative infections, female sex was also associated with higher odds (OR 1.38 [95% CI 1.19–1.59]; p<0.001). Similar to the overall gram-negative cohort, each additional year of age increased the likelihood of monomicrobial gram-negative infection by 3% (OR 1.03 [95% CI 1.02–1.04]; p<0.001). Other significantly associated factors were higher BMI and ASA class, lumbar spinal level, longer duration of surgery, inpatient surgery, surgeries for trauma, and emergency surgeries. Conversely, other gram-positive infections were associated with significantly higher odds of occurring in male patients (OR 1.39 [95% CI 1.06–1.82]; p = 0.019) and lower odds of thoracic spine involvement (OR 0.49 [95% CI 0.32–0.74]; p<0.001) or lumbar spine involvement (OR 0.44 [95% CI 0.32–0.62]; p<0.001) relative to the reference group (Table 4).

**Table 4:** Multinomial logistic regression analysis of predictors of monomicrobial gram-negative and other gram-positive spinal fusion surgical site infections compared with monomicrobial *Staphylococcus aureus* infections in study of microbial etiology of postoperative spinal fusion infections and factors associated with gram-negative infections, United States, 2021–2022*

Characteristic	Monomicrobial gram-negative infections			Other gram-positive infections	
	OR (95% CI)	p value		OR (95% CI)	p value
Age	1.03 (1.02–1.03)	<0.001		1.01 (1.00–1.02)	0.061
BMI	1.03 (1.02–1.04)	<0.001		1.02 (1.00–1.04)	0.043
Male sex	0.73 (0.63–0.84)	<0.001		1.39 (1.06–1.82)	0.019
Outpatient surgery	0.41 (0.18–0.96)	0.040		0.88 (0.29–2.65)	0.8
Duration of surgery	1.20 (1.16–1.24)	<0.001		1.06 (1.00–1.13)	0.066
Surgical approach					
Anterior	Referent			Referent	
Posterior	1.01 (0.80–1.28)	>0.9		0.98 (0.66–1.46)	>0.9
Both	0.86 (0.53-1.39)	0.5		1.31 (0.57–2.99)	0.5
ASA class					
1	Referent			Referent	
2	4.83 (1.05-22.3)	0.044		2.10 (0.27–16.5)	0.5
3	7.04 (1.53-32.3)	0.012		2.33 (0.30–18.1)	0.4
4	15.4 (3.29-72.0)	<0.001		1.96 (0.24–16.3)	0.5
Diabetes				1.20 (0.90–1.60)	0.2
Wound class					
Clean	Referent			Referent	
Clean-contaminated	1.74 (1.06–2.87)	0.029		1.13 (0.43–2.97)	0.8
Contaminated	2.21 (0.66–7.33)	0.2		0.00 (0.00-0.00)	<0.001
Dirty	1.76 (0.80–3.89)	0.2		0.00 (0.00-0.00)	<0.001
Spinal level					
Axis-atlas/cervical	Referent			Referent	
Cervical/dorsal/dorsolumbar	1.11 (0.77–1.61)	0.6		0.59 (0.32–1.08)	0.087
Dorsal/dorsolumbar	1.27 (0.99–1.63)	0.056		0.49 (0.32–0.74)	<0.001
Lumbar/lumbosacral	1.51 (1.22–1.85)	<0.001		0.44 (0.32–0.62)	<0.001
Trauma	1.91 (1.38–2.64)	<0.001		1.04 (0.57–1.89)	0.9
Closure					
Intentionally left open	Referent			Referent	
Fully closed or partial	1.07 (0.50–2.33)	0.9		2.33 (0.30–18.1)	0.4
Timing of infection, days from procedure	0.97 (0.96–0.97)	<0.001		0.99 (0.98–1.00)	0.11
Depth of infection					
Superficial	Referent			Referent	
Deep	0.88 (0.74–1.05)	0.15		0.95 (0.68–1.33)	0.8
Organ space	0.85 (0.70–1.03)	0.10		1.14 (0.79–1.66)	0.5
Emergency	0.99 (0.75–1.31)	>0.9		1.50 (0.93–2.39)	0.093
Use of scope	2.55 (0.49–13.1)	0.3		0.00 (0.00-0.00)	<0.001

*ORs represent the relative odds of monomicrobial gram-negative infections or other gram-positive infection compared with the reference group of monomicrobial *Staphylococcus aureus* spinal fusion surgical site infection. ASA level 5 was removed because of insufficient numbers. ASA, American Society of Anesthesiologists; BMI, body mass index; OR, odds ratio.

## Discussion

Our study represents a large national surveillance-based analysis describing the contemporary microbial etiology of spinal fusion SSI. Our analysis of a national patient cohort showed gram-negative organisms made up nearly one third of all patient cases and organisms identified in diagnostic cultures for spinal fusion SSI. Among gram-negative bacteria, Enterobacterales (most commonly *E. coli*), and *Pseudomonas* spp. were the predominant organisms identified. Gram-negative bacteria have been previously reported to affect 30%–54% of infections, occurring more frequently in infections involving lower spinal levels, particularly lumbar and sacral instrumentation (*5*,*6*). The percentage is much larger than that reported for other orthopedic implant infections, such as periprosthetic joint infection, where gram-negative infection made up ≈11% of cases (*7*).

Consistent with previous literature demonstrating an anatomic gradient wherein lower spinal levels carry an increased likelihood of gram-negative organism involvement in spinal fusion SSI (*8*), our analysis further delineates multiple independent patient-specific and procedural variables that increase the probability of gram-negative pathogen involvement relative to monomicrobial *S. aureus* infection. Specifically, female sex, advanced age, higher BMI, prolonged operative duration, greater comorbidity burden, and emergency and trauma-related procedures were independently associated with a higher likelihood of gram-negative infection compared with infections involving monomicrobial *S. aureus*. The associations remained consistent in a secondary analysis restricted to monomicrobial gram-negative and monomicrobial *S. aureus* infections, demonstrating that the findings are not confounded by coinfecting organisms.

The precise underlying mechanisms driving the observed associations between those independent variables and gram-negative infections remain to be fully elucidated. Of note, our finding that trauma before spinal fusion independently correlates with a gram-negative etiology aligns with existing literature on fracture-related infections, which similarly demonstrate a high proportion of gram-negative infections (*9*). Those parallel clinical observations highlight a critical area for future prospective studies to investigate the specific pathophysiological pathways.

The strengths of this study include the use of a large, national dataset derived from the NHSN, encompassing thousands of spinal fusion SSI cases reported from multiple centers across the United States. This sample size exceeds that of most previously published studies, which have often been limited to single institutions or regional networks and therefore subject to potential biases because of local practice patterns, patient demographics, and institutional protocols. The multicenter nature of the dataset enhances the generalizability of our findings and provides substantially greater statistical power to detect associations among patient characteristics, surgical factors, and microbial etiology.

The first limitation of our study is that the reporting of spinal fusion SSIs to the NHSN is voluntary, introducing the possibility of reporting bias. Because the NHSN database only records infected episodes, the lack of an uninfected control group prevents evaluation of risk factors for development of infection. The use of *S. aureus* infections as the statistical reference limits generalizability because that cohort only involves those that already have infections. Because this national registry dataset contains only standardized, registry-submitted data, we lacked access to granular clinical details, such as specific perioperative antimicrobial protocols, antibiotic susceptibility profiles, use of allografts, instrumentation, and the precise anatomic source of the cultures. Furthermore, the redaction of key geographic and institutional variables, including facility size and type, prevented their inclusion in our multinomial models, limiting our ability to assess the variables that might have been pertinent. As an example, an outbreak of spinal fusion SSIs associated with the use of bone allografts infected with *Mycobacterium tuberculosis* that was reported in 2021 might not be fully captured in our study (*10*).

Because our cohort spans 2021–2022, our findings reflect pandemic-era practice patterns and case mixes. Institutional restrictions and altered clinical priorities during this period might have altered typical patient demographics and surgical volumes. As a consequence, those transient pandemic-era modifications could limit the generalizability of our results to current, postpandemic clinical practice.

In conclusion, gram-negative infections account for nearly one third of spinal fusion SSIs and are associated with specific patient and surgical factors. The identification of independent factors related to gram-negative infections provides a practical foundation for transitioning from a universal approach to risk-stratified clinical decision-making. For researchers, specific high-risk cohorts highlight key target populations for future studies evaluating expanded gram-negative prophylactic regimens. For clinicians, using these risk profiles can guide timely, targeted empiric antimicrobial selection when spinal fusion SSIs occur, ultimately balancing optimal antimicrobial stewardship with tailored patient care.

Appendix Additional information about microbial etiology of postoperative spinal fusion infections and factors associated with gram-negative infections.

## References

[1] Shaw JD, Nelson SB, Simpfendorfer C, Berbari EF, Henry MW, Kleck CJ, et al. New definition for postoperative spine infection (PSI): from the workgroup of the Musculoskeletal Infection Society (MSIS) and the European Bone & Joint Infection Society (EBJIS). Spine. 2025;•••. 10.1097/BRS.0000000000005604 41432018

[2] Edmiston CE Jr, Leaper DJ, Chitnis AS, Holy CE, Chen BPH. Risk and economic burden of surgical site infection following spinal fusion in adults. Infect Control Hosp Epidemiol. 2023;44:88–95. 10.1017/ice.2022.32 35322778

[3] Alfin DJ, Shilong DJ, Bot GM, Dengunu Salun W. Surgical site infection rate in spine surgery, incidence, and risk factors: a ten-year retrospective cohort review in a developing neurosurgical centre. BMC Surg. 2025;25:127. 10.1186/s12893-025-02846-4 40170142 PMC11963446

[4] Centers for Disease Control and Prevention. CDC/NHSN surveillance definitions for specific types of infections, 2024. In: National Healthcare Safety Network Patient Safety Component Manual [cited 2026 Jan 6]. https://www.cdc.gov/nhsn/pdfs/validation/2024/pcsmanual_2024.pdf

[5] Abdul-Jabbar A, Berven SH, Hu SS, Chou D, Mummaneni PV, Takemoto S, et al. Surgical site infections in spine surgery: identification of microbiologic and surgical characteristics in 239 cases. Spine. 2013;38:E1425–31. 10.1097/BRS.0b013e3182a42a68 23873240

[6] Shillingford JN, Laratta JL, Reddy H, Ha A, Lehman RA Jr, Lenke LG, et al. Postoperative surgical site infection after spine surgery: an update from the Scoliosis Research Society (SRS) Morbidity and Mortality Database. Spine Deform. 2018;6:634–43. 10.1016/j.jspd.2018.04.004 30348337

[7] Tai DBG, Patel R, Abdel MP, Berbari EF, Tande AJ. Microbiology of hip and knee periprosthetic joint infections: a database study. Clin Microbiol Infect. 2022;28:255–9. 10.1016/j.cmi.2021.06.006 34129907 PMC8665939

[8] Long DR, Bryson-Cahn C, Pergamit R, Tavolaro C, Saigal R, Chan JD, et al. 2021 Young investigator award winner: anatomic gradients in the microbiology of spinal fusion surgical site infection and resistance to surgical antimicrobial prophylaxis. Spine. 2021;46:143–51. 10.1097/BRS.0000000000003603 32796459 PMC8299899

[9] Depypere M, Sliepen J, Onsea J, Debaveye Y, Govaert GAM, IJpma FFA, et al. The microbiological etiology of fracture-related infection. Front Cell Infect Microbiol. 2022;12:934485. 10.3389/fcimb.2022.934485 35873162 PMC9300981

[10] Wortham JM, Haddad MB, Stewart RJ, Annambhotla P, Basavaraju SV, Nabity SA, et al. Second nationwide tuberculosis outbreak caused by bone allografts containing live cells—United States, 2023. MMWR Morb Mortal Wkly Rep. 2024;72:1385–9. 10.15585/mmwr.mm725253a1 38175804

