# Supplementary material for: Microbial Etiology of Postoperative Spinal Fusion Infections and Factors Associated with Gram-Negative Infections, United States, 2021–2022
Source: Emerg Infect Dis. 2026 Nov;32(11):1838–45. doi: 10.3201/eid3211.260662 (PMC13715884; doi:10.3201/eid3211.260662)
Supplement: Supplementary file 1 [file 26-0662-Techapp-s1.pdf]

Article DOI: https://doi.org/10.3201/eid3211.260662

EID cannot ensure accessibility for supplementary materials supplied by authors. Readers who have difficulty accessing supplementary content should contact the authors for assistance.

Microbial Etiology of Postoperative Spinal Fusion Infections and Factors Associated with Gram-Negative Infections, United States, 2021–2022

Appendix

Appendix Table. Pathogens Associated with Spinal Fusion Surgical Site Infections

Organism	Count
Achromobacter xylosoxidans	3
Acinetobacter	6
Acinetobacter baumannii	20
Acinetobacter calcoaceticus	2
Acinetobacter calcoaceticus-baumannii complex	4
Acinetobacter lwoffi	1
Acinetobacter pittii	3
Acinetobacter radioresistens	4
Actinomyces	4
Actinomyces israelii	1
Actinomyces neuii	2
Actinomyces odontolyticus	1
Actinomyces radingae	1
Actinotignum schaalii	2
Aerococcus urinae	1
Aeromonas	1
Aeromonas hydrophila complex	2
Anaerobe	3
Anaerobic Gram-negative bacillus	1
Anaerobic Gram-positive bacillus	3
Anaerobic Gram-positive coccus	6
Anaerococcus	3
Anaerococcus vaginalis	2
Aspergillus fumigatus	2
Bacillus	10
Bacillus cereus	3
Bacillus cereus group	2
Bacillus, not B. anthracis	4
Bacteroides	4
Bacteroides caccae	1
Bacteroides fragilis	39
Bacteroides fragilis group	8
Bacteroides ovatus	7
Bacteroides thetaiotaomicron	8
Bacteroides vulgatus	2
Beta-hemolytic *streptococcus*	13
Beta-hemolytic *Streptococcus,* group A	3
Beta-hemolytic *streptococcus,* non-Group A, non-Group B	2
Bipolaris	1

Organism	Count
Burkholderia cepacia	1
Candida albicans	110
Candida auris	2
Candida dubliniensis	5
Candida glabrata	16
Candida guilliermondii	1
Candida lusitaniae	1
Candida metapsilosis	1
Candida orthopsilosis	1
Candida parapsilosis	21
Candida tropicalis	6
Capnocytophaga	1
Chryseomonas luteola	1
Citrobacter	1
Citrobacter amalonaticus	1
Citrobacter braakii	4
Citrobacter freundii	24
Citrobacter koseri	37
Cladosporium	4
Clostridium bifermentans	3
Clostridium perfringens	3
Coliform bacteria	2
Corynebacterium	47
Corynebacterium amycolatum	7
Corynebacterium coyleae	3
Corynebacterium glucuronolyticum	2
Corynebacterium jeikeium	4
Corynebacterium striatum	43
Corynebacterium tuberculostearicum	4
Corynebacterium, not C. jeikeium	2
Curtobacterium	3
Curtobacterium albidum	1
Cutibacterium	39
Cutibacterium acnes	242
Diphtheroids	29
Diphtheroids, aerobic	1
Enterobacter	5
Enterobacter amnigenus	1
Enterobacter asburiae	2
Enterobacter cloacae	129
Enterobacter cloacae complex	155
Enterobacter hormaechei	3
Enterococcus	25
Enterococcus avium	6
Enterococcus faecalis	253
Enterococcus faecium	75
Enterococcus gallinarum	2
Enteropathogenic *Escherichia coli*	1
Escherichia coli	579
Escherichia vulneris	1
Finegoldia magna	35
Fungi	1
Fusobacterium nucleatum	4
Globicatella sanguinis	1
Gram-negative bacillus	17
Gram-negative bacillus, fermentative	2
Gram-positive bacillus	8
Gram-positive coccus	23
Granulicatella adiacens	5
Granulicatella elegans	1
Hemophilus	1
Hemophilus parainfluenzae	8
Hafnia alvei	3
Klebsiella	10
Klebsiella aerogenes	81
Klebsiella oxytoca	68
Klebsiella pneumoniae	281
Klebsiella pneumoniae pneumoniae	27
Klebsiella variicola	5

Organism	Count
Kocuria	1
Lactobacillus	11
Lactobacillus casei	1
Leclercia adecarboxylata	1
Micrococcus luteus	4
Morganella morganii	87
Mycobacterium	2
Mycobacterium avium	1
Mycobacterium chelonae	1
Mycobacterium fortuitum	3
Mycobacterium smegmatis	1
Mycobacterium tuberculosis complex	1
Mycoplasma hominis	4
Neisseria	1
Ochrobactrum anthropi	1
Paenibacillus provencensis	1
Pantoea	6
Pantoea agglomerans	4
Parabacteroides distasonis	1
Pasteurella multocida multocida	1
Peptoniphilus	3
Peptoniphilus asaccharolyticus	7
Peptoniphilus harei	4
Peptostreptococcus	9
Prevotella	15
Prevotella bivia	18
Prevotella disiens	3
Prevotella intermedia	1
Prevotella melaninogenica	3
Propionibacterium	21
Propionibacterium avidum	5
Proteus	4
Proteus hauseri	1
Proteus mirabilis	367
Proteus penneri	5
Proteus vulgaris	21
Providencia rettgeri	4
Providencia stuartii	3
Pseudomonas	6
Pseudomonas aeruginosa	523
Pseudomonas fluorescens	1
Pseudomonas mendocina	1
Pseudomonas putida	2
Pseudomonas stutzeri	2
Raoultella ornithinolytica	3
Rhodotorula rubra	1
Rothia mucilaginosa	1
Salmonella	1
Serratia	3
Serratia liquefaciens	1
Serratia marcescens	160
Small-colony-forming β-hemolytic group C streptococci	3
Staphylococcus	39
Staphylococcus aureus	2275
Staphylococcus capitis	23
Staphylococcus capitis capitis	2
Staphylococcus caprae	4
Staphylococcus cohnii	2
Staphylococcus epidermidis	536
Staphylococcus hemolyticus	15
Staphylococcus hominis	18
Staphylococcus intermedius	5
Staphylococcus kloosii	1
Staphylococcus lugdunensis	73
Staphylococcus pettenkoferi	1
Staphylococcus pseudintermedius	11
Staphylococcus saccharolyticus	7
Staphylococcus saprophyticus	2
Staphylococcus saprophyticus saprophyticus	1

Organism	Count
Staphylococcus schleiferi	5
Staphylococcus sciuri	1
Staphylococcus simulans	3
Staphylococcus warneri	6
Staphylococcus, coagulase negative	246
Staphylococcus, coagulase positive	3
Stenotrophomonas maltophilia	6
Streptococcus	12
Streptococcus agalactiae (group B streptococci)	63
Streptococcus anginosus	18
Streptococcus anginosus group	3
Streptococcus constellatus	10
Streptococcus constellatus constellatus	1
Streptococcus dysgalactiae	11
Streptococcus dysgalactiae dysgalactiae	1
Streptococcus dysgalactiae equisimilis	5
Streptococcus gordonii	1
Streptococcus intermedius	12
Streptococcus mitis	2
Streptococcus mitis group	3
Streptococcus oralis	1
Streptococcus parasanguinis	2
Streptococcus pneumoniae	1
Streptococcus pyogenes (group A streptococci)	14
Streptococcus salivarius	1
Streptococcus sanguinis	2
Streptococcus, group C	5
Streptococcus, group G	1
Veillonella	2
Veillonella parvula	4
Viridans group streptococci	8
Yeast	13
Abiotrophia	1
Abiotrophia defectiva	1
Acinetobacter ursingii	2
Actinomyces europaeus	1
Alpha-hemolytic *streptococcus*	3
Arcanobacterium hemolyticum	1
Bacteroides pyogenes	1
Brevundimonas	1
Campylobacter ureolyticus	1
Citrobacter werkmanii	1
Clostridium innocuum	1
Corynebacterium propinquum	1
Dermabacter hominis	1
Eggerthella lenta	1
Eikenella	1
Eikenella corrodens	1
Enterobacter cancerogenus	1
Enterococcus casseliflavus	2
Enterococcus raffinosus	2
Finegoldia	1
Gamma (non)-hemolytic *streptococcus*	1
Micrococcus	2
Oligella urethralis	1
Pluralibacter gergoviae	1
Prevotella buccae	1
Prevotella denticola	2
Prevotella oris	1
Propionibacterium granulosum	1
Raoultella	1
Rothia	1
Staphylococcus auricularis	1
Staphylococcus xylosus	1
Stenotrophomonas	1
Veillonella dispar	1
Actinomyces meyeri	1
Clostridium cadaveris	1
Corynebacterium aurimucosum	2

Organism	Count
Corynebacterium kroppenstedtii	1
Corynebacterium minutissimum	1
Fusobacterium	1
Fusobacterium necrophorum	1
Moraxella osloensis	1
Neisseria oralis	1
Peptostreptococcus anaerobius	2
Porphyromonas somerae	2
Staphylococcus hominis hominis	2
Staphylococcus pasteuri	1
Streptococcus gallolyticus	1
